# Towards high-resolution *in-situ* structural biology of membrane protein complexes

**DOI:** 10.1063/4.0001077

**Published:** 2025-10-27

**Authors:** Rilee Zeinert, Madolyn Britt, Elissa Moller, Fei Zhou, Alexander Sodt, Gisela Storz, Sergei Sukharev, Doreen Matthies

**Affiliations:** 1 Eunice Kennedy Shriver National Institute of Child Health and Human Development, National Institutes of Health, Bethesda, MD, USA; 2 Eunice Kennedy Shriver National Institute of Child Health and Human Development, National Institutes of Health, Bethesda, MD, USA; 3 Department of Biology, University of Maryland, College Park, MD, USA

## Abstract

Membrane protein structure determination is technically challenging and further complicated by the removal or displacement of lipids, which can result in complex dissociation, non-native conformations, or a strong preference for certain states at the exclusion of others. Here, we will showcase two examples where we use mild membrane protein extraction methods, followed by single-particle cryo-EM to reveal more native high- resolution structural information of membrane protein complexes. (1) P-type ATPases have been structurally characterized as monomers after being extracted from biological membranes using detergents. Here, we use a variety of detergents and polymers to extract and characterize magnesium transporter P-type ATPase MgtA from Escherichia coli and find that the protein exists and functions as a dimer when extracted using mild detergents or polymers. We obtained high-resolution cryo-EM structures of the homodimeric form, which shed light on the dimer interface, ion-binding sites as well as the predicted unstructured N-terminal tail, which is well resolved in our dimeric cryo-EM maps. (2) E. coli MscS, a model system for MSC gating, is an inner membrane protein that opens when external osmolarity changes cause water influx and stretches the membrane. The efflux of osmolytes through these channels reduces the osmotic gradient and prevents cell lysis, enabling bacteria to colonize osmotically challenging host environments and survive transmission through fresh water. As a tension sensor, MscS is very sensitive and highly adaptive. It readily opens under super-threshold tension and closes upon tension reduction, but under lower tensions, it slowly inactivates and can only recover after tension release. Existing cryo-EM structures do not explain the entire functional gating cycle of open, closed, and inactivated states. A central question in the field has been the assignment of the frequently observed non-conductive conformation to either a closed or inactivated state. Here, we solved a 3 Å cryo-EM structure of MscS in native nanodiscs obtained via extraction with the novel Glyco- DIBMA polymer, eliminating the detergent solubilization and lipid removal step common to all prior structures. We observe densities of endogenous phospholipids between the transmembrane helices, stabilized by electrostatics interactions. Through mutations we examine the functional effects of their destabilization, illustrating a novel lipid-mediated inactivation mechanism based on an uncoupling of the peripheral tension- sensing helices from the gate. The use of this polymer increased the predictive power of our cryo-EM structure, allowing us to associate the solved conformation with the inactivated state of the multi-state MSC MscS.

